# Estimation of true height: a study in population-specific methods among young South African adults

**DOI:** 10.1017/S1368980016002330

**Published:** 2016-09-09

**Authors:** Christen Renée Lahner, Susanna Maria Kassier, Frederick Johannes Veldman

**Affiliations:** University of KwaZulu-Natal, Dietetics and Human Nutrition, School of Agricultural, Earth and Environmental Sciences, College of Agriculture, Engineering and Science, Pietermaritzburg, KwaZulu-Natal, South Africa

**Keywords:** Anthropometry, Arm-associated height estimationmethods, Population-specific methodology, Vitruvius theory, Maximal height theory

## Abstract

**Objective:**

To investigate the accuracy of arm-associated height estimation methods in the calculation of true height compared with stretch stature in a sample of young South African adults.

**Design:**

A cross-sectional descriptive design was employed.

**Setting:**

Pietermaritzburg, Westville and Durban, KwaZulu-Natal, South Africa, 2015.

**Subjects:**

Convenience sample (*N* 900) aged 18–24 years, which included an equal number of participants from both genders (150 per gender) stratified across race (Caucasian, Black African and Indian).

**Results:**

Continuous variables that were investigated included: (i) stretch stature; (ii) total armspan; (iii) half-armspan; (iv) half-armspan ×2; (v) demi-span; (vi) demi-span gender-specific equation; (vii) WHO equation; and (viii) WHO-adjusted equations; as well as categorization according to gender and race. Statistical analysis was conducted using IBM SPSS Statistics Version 21.0. Significant correlations were identified between gender and height estimation measurements, with males being anatomically larger than females (*P*<0·001). Significant differences were documented when study participants were stratified according to race and gender (*P*<0·001). Anatomical similarities were noted between Indians and Black Africans, whereas Caucasians were anatomically different from the other race groups. Arm-associated height estimation methods were able to estimate true height; however, each method was specific to each gender and race group.

**Conclusions:**

Height can be calculated by using arm-associated measurements. Although universal equations for estimating true height exist, for the enhancement of accuracy, the use of equations that are race-, gender- and population-specific should be considered.

For the assessment of true height, an individual is required to be in a compos mentis state and should be able stand up straight unaided without constraints such as medical equipment, physical disabilities and/or space limitations^(^ ^1^ ^–^ ^3^ ^)^. However, certain environments serve as a barrier to such an idealistic situation^(^ ^2^ ^)^. As a result, body-part measurements are used in the calculation of estimated height^(^ ^1^ ^,^ ^3^ ^)^. Arm-associated height estimation methods are more commonly used as arm long bones are not affected by ageing^(^ ^4^ ^)^.

Height or estimations thereof are useful in various settings, as an accurate height measurement forms part of the BMI equation ([weight (kg)]/[height (m)]^2^) to evaluate weight status^(^ ^5^ ^)^. In addition, accurate height measurements are essential when determining resting energy expenditure or BMR^(^ ^3^ ^)^ as calculated for inclusion in the Harris–Benedict equation^(^ ^6^ ^)^, for spirometry^(^ ^3^ ^,^ ^7^ ^)^, calculating cardiac function indices^(^ ^8^ ^)^, drug dose adjustments^(^ ^8^ ^)^ and the creatinine height index^(^ ^3^ ^)^. Therefore, use of an inaccurate height measurement *v*. an accurate one may have adverse outcomes (see online supplementary material, Supplemental Fig. 1).

Inaccurate anthropometric values can occur when the measurement technique used was not validated for the population in question *v*. the study population for which the equation was developed or if technical errors of measurement occurred^(^ ^9^ ^)^. Inaccurate height measurements result in the inaccurate calculation and misinterpretation of an individual’s nutritional status and hence nutrient requirements^(^ ^10^ ^)^. This can lead to overfeeding^(^ ^10^ ^)^ or underfeeding^(^ ^10^ ^)^. Underfeeding initiates the malnutrition cycle, which in turn increases the risk for medical complications such as poor wound healing, nosocomial infections, organ failure and increased length of hospital stay. Overfeeding, on the other hand, is associated with increased risk for lipogenesis, hyperglycaemia and respiratory failure, which can affect morbidity and mortality. These potential consequences emphasize the need for clinicians to choose anthropometric methods that are population-specific^(^ ^11^ ^)^.

The present paper reports the outcomes of a study that aimed to determine the accuracy of arm-associated height estimation methods in the calculation of true height compared with stretch stature in a sample of young South African adults and, where relevant, allude to possible adjustments to existing height estimation equations.

## Methods

### Study design

A cross-sectional descriptive survey was conducted with the study sample consisting of a convenience sample of young South African adults. Prior to data collection, a pilot study^(^ ^11^ ^)^ was conducted with the purpose of investigating the plausibility of the research question as well as the standardization of anthropometric measurement techniques between fieldworkers.

### Study setting

The study setting was Pietermaritzburg, Westville and Durban, in KwaZulu-Natal, South Africa, 2015.

### Participants

Participants from various self-reported race groups were conveniently sampled in a stratified manner in order to include 150 males and 150 females aged 18–24 years from each of the following race groups: (i) Black Africans; (ii) Caucasians; and (iii) Indians, all of which typically inhabit KwaZulu-Natal and South Africa at large^(^ ^12^ ^)^. The above yielded a study sample of *N* 900 participants.

### Convenience sampling method

Data collection stations were set up in high-traffic areas within the campus grounds of the University of KwaZulu-Natal (UKZN). Individuals who were in high proximity to these stations were recruited for participation in the study according to the inclusion/exclusion criteria. Specifically, exclusion criteria consisted of physical disabilities or amputations, as that would prevent participants from standing unaided. In order to be included in the present study, the participants were required to be: (i) registered for study at UKZN; (ii) aged 18–24 years; (iii) Caucasian, Black African or Indian; and (iv) born in South Africa and be in possession of a South African identity document.

### Fieldworkers

Trained fieldworkers measured anthropometric measurements according to the International Society for the Advancement of Kinanthropometry (ISAK) standards. All fieldworkers were registered for study in dietetics or human nutrition at the time of the study.

### Data collection stations

Observers set up mobile data collection stations erected in close proximity to an even surfaced wall with a 90° angle level to an even surfaced floor. There were a minimum of three fieldworkers at each data collection station at any given time. In addition, the data collection was specifically planned to take place during February and March 2015 (late summer), as this would allow participants to be more likely to agree to removing shoes, socks and outer layers of clothing such as jackets and jerseys, to facilitate the accurate measurement of standing height.

### Height measurement

#### Stretch stature

Stretch stature (SS) is the gold standard of measuring true height^(^ ^13^ ^)^. The instrument used to measure SS was a portable stadiometer (Seca) with a sliding head board, that was manufactured according to ISAK standards^(^ ^13^ ^)^. The height measurements were repeated three times to one decimal place and the mean of two closest values calculated. The procedure for measuring height was as follows:

1.
The stadiometer was placed on a level even surface and against a wall that was at a 90° angle to the floor.
2.
Participants were asked to remove shoes, socks and outer layers of clothing such as a jacket.
3.
Participants were asked to untie their hair if it was tied up. In the case of traditional hairstyles such as braids and weaves typically worn by Black African women, a knitting needle was inserted gently through the hairstyle in order to give an indication of where the skull was. Then a ruler was used to accurately appraise height by reading it from the stadiometer.
4.
Participants were then asked to stand on the foot board of the stadiometer, positioned as follows: (i) the participant facing outwards, with his/her (ii) hands placed to his/her sides, (iii) legs straight, (iv) feet together and (v) head in the Frankfort plane (see online supplementary material, Supplemental Fig. 2). The Frankfort plane is achieved when the orbitale is in the same horizontal plane as the tragion.
5.
The back of the heels, buttocks and upper back had to be touching the stadiometer.
6.
The first observer (observer A) placed one hand on either side of the participant’s jawline and applied ‘upward pressure’ into the mastoid processes. The same observer asked the participant to inhale and hold his/her breath.
7.
The second observer (observer B) placed the sliding head board on to the vertex of the participant and read the height measurement out loud to the third observer (observer C) who recorded the height (in centimetres) to one decimal place. When reading the measurement, observer B called out each measurement one at a time. For example, 186·2 cm would be called out as one, eight, six, point, two.

### Arm-associated height measurements

Arm-associated height estimation measurements were taken for all participants. These included: (i) total armspan (TAS); (ii) half-armspan (HAS); and (iii) demi-span (DS; see online supplementary material, Supplemental Fig. 3). Each measurement was taken three times and recorded to the nearest 0·1 cm. The mean of the two closest values was recorded.

#### Total armspan

The TAS measurement^(^ ^14^ ^)^ was taken using a stainless steel measuring tape, calibrated in centimetres. The following method was used:

1.
The participant was asked to remove outermost layers of clothing such as a jacket, as it could restrict arm movement.
2.
The participant was then asked to stand against the wall, with: (i) his/her arms outstretched in a horizontal plane in relation to the floor, (ii) palms facing outwards and (iii) the arms placed at a 90° angle to the participant’s body.
3.
The 90° angle was verified using a triangle protractor.
4.
Observer A and observer B stood on either side of the participant, while observer C recorded the measurement (see online supplementary material, Supplemental Fig. 4).
5.
The stainless steel measuring tape was used to measure TAS from the right dactylion to the left dactylion.

#### Half-armspan

The HAS measurement^(^ ^2^ ^,^ ^15^ ^)^ was taken using a stainless steel measuring tape, calibrated in centimetres. The measurements were taken on the right hand-side of the body in accordance with ISAK standards^(^ ^13^ ^)^. The following method was used:

1.
The participant was asked to remove outermost layers of clothing such as a jacket, as it could restrict arm movement.
2.
The participant was then asked to stand against the wall, with: (i) his/her right arm outstretched in a horizontal plane in relation to the floor, (ii) the palm facing outwards and (iii) the arm placed at a 90° angle to the participant’s body.
3.
The 90° angle was verified using a triangle protractor.
4.
Observer A stood at the suprasternal notch while observer B stood on right side of the participant. Observer C recorded the measurement (see online supplementary material, Supplemental Fig. 5).

#### Demi-span

The DS is measured from the suprasternal notch to the proximal digital crease of the middle finger. For the purpose of the current study, DS was calculated by measuring the phalanx distance (see online supplementary material, Supplemental Fig. 6). Phalanx distance was measured from the right dactylion to the proximal digital crease of the middle finger. This measurement was then subtracted from the HAS measurement. A ruler, calibrated in centimetres, was used to measure phalanx distance. The measurements were conducted on the right-hand side of the body in accordance with ISAK standards^(^ ^13^ ^)^. The original method for measuring DS^(^ ^16^ ^)^ takes the measurement directly (see online supplementary material, Supplemental Fig. 7).

#### Correct positioning

The positioning of the hand, wrist and arm are vital for the accurate measurement of arm-associated measurements. For the purpose of the current study, guidelines were developed to identify correct positioning. Hence, the following method was used:

1.
The wrist was in the neutral A position (see online supplementary material, Supplemental Fig. 8). The participant was asked to avoid extension and flexion movements in the wrist. In this context, extension refers to negative angle movements while flexion refers to positive angle movements.
2.
The wrist was in the neutral B position (see online supplementary material, Supplemental Fig. 9). The participant was asked to avoid radial and ulnar deviation movements in the wrist.
3.
The arm was extended at a 90° angle to the body, with elbows straightened.
4.
The participant was asked to place his/her fingers together (small, ring, middle and index fingers together) with the thumb pointing upwards.

### Equations

The measurements obtained from data collection were entered into two equations, namely the demi-span equation (DSE)^(16)^ and the WHO equation^(^ ^17^ ^)^. The equations were applied accordingly and values were calculated to one decimal place.

DSE:

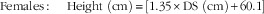




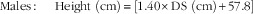




WHO equation:

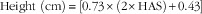




### Statistical analysis

For the purpose of the current study, the continuous variables included: (i) SS (dependent); (ii) TAS; (iii) HAS; (iv) HAS×2; (v) DS; (vi) DSE; (vii) WHO equation; and (viii) WHO-adjusted equation; as well as categorization according to gender and race. Statistical analysis was conducted using the statistical software package IBM SPSS Statistics Version 21.0. The significance level (*α*) adopted in the statistical analysis was *P*<0·05. The statistical tests that were applied included: (i) descriptive statistics, describing the characteristics of the study sample; (ii) the *χ* ^2^ test, investigating the comparison between categories; (iii) the Pearson correlation coefficient, identifying the strength of association between variables; (iv) the one-sample *t* test, investigating the comparison between two continuous variables; (v) the independent-samples *t* test, investigating the comparison between two categories within the same variable; and (vi) the Bland–Altman strategy, to measure agreement between measurements.

## Results

The mean age of the study sample was 20·3 (sd 1·7) years. The mean SS of the race groups, according to gender, was: Caucasian males, 179·3 (sd 6·7) cm; Caucasian females, 165·9 (sd 6·8) cm; Indian males, 172·0 (sd 6·5) cm; Indian females, 159·0 (sd 5·9) cm; Black African males, 170·5 (sd 6·6) cm; Black African females, 158·7 (sd 6·1) cm.

SS was statistically significantly correlated (*P*<0·001; see online supplementary material, Supplemental Table 1) with all of the arm-associated height estimation measurements. TAS and SS were most strongly correlated (*r*=0·887; *P*<0·001), whereas the correlation with the WHO-adjusted equation was the weakest (*r*=0·186; *P*<0·001). Statistically significant differences (*P*<0·001) were found for all height estimation methods when compared between genders.

Across all height estimation measurements, there were no significant differences when comparing Black Africans and Indians. Statistically significant differences (*P*<0·001) across all height estimation measurements were, however, measured between the Caucasian and Indian groups as well as between the Black African and Caucasian groups.

When the race groups were compared by gender, all height estimation measurements between Caucasian and Indian males were significantly different (*P*<0·001; Table 1), except for TAS (mean difference (MD)=2·6 cm; *P*<0·05). Significant differences (*P*<0·001) were identified across all height estimation measurements when comparing Black African and Caucasian males. The comparison between Black African and Indian males rendered significant differences only for SS (*P*<0·05) and the WHO-adjusted equation (*P*<0·001).

**Table 1:** Comparison of height estimation measurements according to race among a convenience sample of young South African adult males aged 18–24 years, KwaZulu-Natal, 2015

	Black African males (*n* 150) *v*. Caucasian males (*n* 150)						Caucasian males (*n* 150) *v*. Indian males (*n* 150)						Black African males (*n* 150) *v*. Indian males (*n* 150)					
Variable	Race	Mean (cm)	Median (cm)	sd (cm)	MD (cm)	*P* value*	Race	Mean (cm)	Median (cm)	sd (cm)	MD (cm)	*P* value*	Race	Mean (cm)	Median (cm)	sd (cm)	MD (cm)	*P* value*
SS	B	170·5	171·1	6·6	−8·7	<0·001	C	179·3	179·0	6·7	7·3	<0·001	B	170·5	171·1	6·6	−1·5	0·05
	C	179·3	179·0	6·7			I	172·0	171·5	6·5			I	172·0	171·5	6·5		
TAS	B	177·9	178·8	7·7	−4·0	<0·001	C	182·0	181·6	8·0	2·6	0·005	B	177·9	178·8	7·7	−1·4	0·126
	C	182·0	181·6	8·0		<0·001	I	179·3	178·2	8·2			I	179·3	178·2	8·2		
DS	B	81·6	81·9	3·6	−1·6	<0·001	C	83·2	83·1	4·1	1·6	0·001	B	81·6	81·9	3·6	−0·0	0·994
	C	83·2	83·1	4·1		<0·001	I	81·6	81·5	4·2			I	81·6	81·5	4·2		
DSE	B	172·0	172·5	5·1	−2·2	<0·001	C	174·3	174·2	5·8	2·2	0·001	B	172·0	172·5	5·0	−0·0	0·994
	C	174·3	174·2	5·8		<0·001	I	172·0	171·9	5·9			I	172·0	171·9	5·9		
HAS	B	89·7	90·0	3·8	−1·7	<0·001	C	91·4	91·4	4·3	1·6	0·001	B	89·7	90·0	3·8	−0·1	0·852
	C	91·4	91·4	4·3		<0·001	I	89·8	89·5	4·5			I	89·8	89·5	4·5		
HAS×2	B	179·4	180·0	7·7	−3·5	<0·001	C	182·9	182·9	8·6	3·3	0·001	B	179·4	180·0	7·7	−0··2	0·852
	C	182·9	182·9	8·6		<0·001	I	179·6	179·0	9·0			I	179·6	179·0	9·0		
WHO equation	B	131·4	131·8	5·6	−2·5	<0·001	C	133·9	133·9	6·3	2·4	0·001	B	131·4	131·8	5·6	−0·1	0·852
	C	133·9	133·9	6·3		<0·001	I	131·5	131·1	6·6			I	131·5	131·1	6·6		
WHO-adjusted	B	170·5	171·0	5·6	11·8	<0·001	C	158·7	158·9	5·7	−20·5	<0·001	B	170·5	171·0	5·6	−8·7	<0·001
equation	C	158·7	158·9	5·7			I	179·3	179·3	6·3			I	179·3	179·3	6·3		

MD, mean difference; SS, stretch stature; TAS, total armspan; DS, demi-span; DSE, demi-span gender-specific equation; HAS, half-armspan; B, Black African; C, Caucasian; I, Indian.

* Independent-samples *t* test. Difference is significant at the 0·05 level.

All differences in the height estimation measurements between Indian and Caucasian females were significant (*P*<0·001; Table 2). When comparing Black African with Indian females, significant differences (*P*<0·05) were found for DS (MD=0·8 cm), DSE (MD=1·1 cm), HAS (MD=0·9 cm), HAS×2 (MD=1·8 cm), the WHO equation (MD=1·3 cm) and the WHO-adjusted equation (MD=6·8 cm; *P*<0·001). There was no significant difference for TAS (MD=1·1 cm) and SS (MD=−0·3 cm). In contrast, comparing Black African and Caucasian females, significant differences (*P*<0·001) were found for SS (MD=−7·1 cm) and the WHO-adjusted equation (MD=−6·1 cm). Significant differences were also identified for TAS (MD=−1·8; *P*<0·05). However, no differences were identified for DS (MD=−0·8 cm), DSE (MD=−1·0), HAS (MD=−0·8 cm) and HAS×2 (MD=−1·5 cm).

**Table 2:** Comparison of height estimation measurements according to race among a convenience sample of young South African adult females aged 18–24 years, KwaZulu-Natal, 2015

	Black African females (*n* 150) *v*. Caucasian females (*n* 150)						Caucasian females (*n* 150) *v*. Indian females (*n* 150)						Black African females (*n* 150) *v*. Indian females (*n* 150)					
Variable	Race	Mean (cm)	Median (cm)	sd (cm)	MD (cm)	*P* value*	Race	Mean (cm)	Median (cm)	sd (cm)	MD (cm)	*P* value*	Race	Mean (cm)	Median (cm)	sd (cm)	MD (cm)	*P* value*
SS	B	158·7	159·0	6·1	−7·1	<0·001	C	165·9	165·9	6·7	6·8	<0·001	B	158·7	159·0	6·1	−0·3	0·663
	C	165·9	165·9	6·7			I	159·0	158·9	5·9			I	159·0	159·9	5·9		
TAS	B	164·2	165·1	8·1	−1·8	0·045	C	166·0	165·8	7·7	3·0	0·001	B	164·2	165·1	8·1	1·1	0·198
	C	166·0	165·8	7·7			I	163·1	162·4	7·1			I	163·1	162·4	7·1		
DS	B	75·4	75·2	3·6	−0·8	0·073	C	76·2	76·1	3·8	1·6	<0·001	B	75·4	75·2	3·6	0·8	0·048
	C	76·2	76·1	3·8			I	74·6	74·4	3·5		<0·001	I	74·6	74·4	3·5		
DSE	B	161·9	161·6	4·9	−1·0	0·073	C	163·0	162·8	5·1	2·1	<0·001	B	161·9	161·6	4·9	1·1	0·048
	C	163·0	162·8	5·1			I	160·8	160·6	4·7		<0·001	I	160·8	160·6	4·7		
HAS	B	83·0	83·1	3·9	−0·8	0·098	C	83·8	83·7	4·1	1·7	<0·001	B	83·0	83·1	3·9	0·9	0·039
	C	83·8	83·7	4·1			I	82·1	82·0	3·7		<0·001	I	82·1	82·0	3·7		
HAS×2	B	166·0	166·2	7·7	−1·5	0·098	C	167·5	167·5	8·1	3·3	<0·001	B	166·0	166·2	7·7	1·8	0·039
	C	167·5	167·5	8·1			I	164·2	164·0	7·4		<0·001	I	164·2	164·0	7·4		
WHO equation	B	121·6	121·8	5·6	−1·1	0·098	C	122·7	122·7	5·9	2·4	<0·001	B	121·6	121·8	5·6	1·3	0·039
	C	122·7	122·7	5·9			I	120·3	120·1	5·4		<0·001	I	120·3	120·1	5·4		
WHO-adjusted	B	165·9	165·8	5·9	−6·1	<0·001	C	172·0	171·6	6·6	13·0	<0·001	B	165·9	165·8	5·9	6·8	<0·001
equation	C	172·0	171·6	6·6			I	159·0	158·9	5·4			I	159·0	158·9	5·4		

MD, mean difference; SS, stretch stature; TAS, total armspan; DS, demi-span; DSE, demi-span gender-specific equation; HAS, half-armspan; B, Black African; C, Caucasian; I, Indian.

* Independent-samples *t* test. Difference is significant at the 0·05 level.

**Fig. 1:**
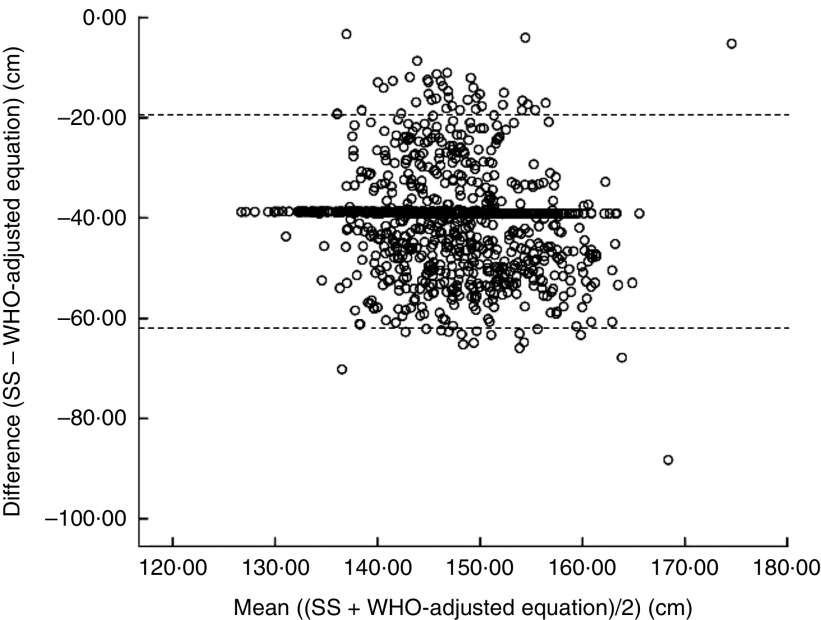
Bland–Altman plot assessing the agreement between stretch stature (SS) and the WHO-adjusted equation for estimating true height in a convenience sample (*N* 900) of young South African adults aged 18–24 years that included equal numbers both genders stratified across race groups (Caucasian, Black African and Indian), KwaZulu-Natal, 2015. The difference between SS and the WHO-adjusted equation is plotted *v*. the mean of the two methods, where – – – – – represent the limits of agreement

Across all race and gender groups, there was a significant difference (*P*<0·001) between SS and the WHO equation. The WHO equation significantly (*P*<0·001) underestimated true height. Based on the mean differences identified between SS and the WHO equation, the WHO equation was adjusted to develop the WHO-adjusted equations (see online supplementary material, Supplemental Table 2). Figure 1 compares the mean differences between SS and the WHO-adjusted equations as a Bland–Altman plot, which demonstrated a strong agreement between the two methods.

Table 3 shows the differences between SS and height estimation measurements using the various methods and their comparison by gender and race. SS minus arm-associated height estimates were significantly different (*P*<0·001) by gender, regardless of race. However, when race was taken into consideration, similarities as well as variations were documented.

**Table 3:** Difference between stretch stature and height estimation measurements according to race and gender among a convenience sample of young South African adults aged 18–24 years, KwaZulu-Natal, 2015

		SS minus TAS						SS minus DSE						SS minus HAS×2						SS minus WHO-adjusted equation					
Race	Gender	Mean (cm)	Median (cm)	sd (cm)	MD (cm)	*P* value*	*P* value†	Mean (cm)	Median (cm)	sd (cm)	MD (cm)	*P* value*	*P* value†	Mean (cm)	Median (cm)	sd (cm)	MD (cm)	*P* value*	*P* value†	Mean (cm)	Median (cm)	sd (cm)	MD (cm)	*P* value*	*P* value†
B, C & I	M	−5·8	−5·9	4·9	−2·6	–	<0·001	1·1	0·6	5·4	1·8	–	<0·001	−6·7	−7·0	6·1	−2·0	–	<0·001	4·4	1·1	13·9	8·8	–	<0·001
(*N* 900)	F	−3·2	−3·5	4·9				−0·7	−0·9	4·6			<0·001	−4·7	−5·0	5·1			<0·001	−4·4	−3·0	8·1			<0·001
B (*n* 150)	M	−7·4	−7·0	4·2	−4·7	<0·001	<0·001	−1·5	−1·34	4·0	−6·5	<0·001	<0·001	−8·9	−8·5	4·4	−5·3	<0·001	<0·001	0·0	0·0	3·7	20·5	0·999	<0·001
C (*n* 150)		−2·7	−2·8	5·0		<0·001	<0·001	5·0	4·6	5·6		<0·001	<0·001	−3·6	−4·2	6·8		<0·001	<0·001	20·5	21·3	8·7		<0·001	
B (*n* 150)	F	−5·4	−5·2	4·3	−5·3	<0·001	<0·001	−3·2	−3·1	3·5	−6·1	<0·001	<0·001	−7·3	−6·7	5·4	−6·1	<0·001	<0·001	−7·1	−6·5	8·8	−1·0	<0·001	0·322
C (*n* 150)		−0·1	−0·8	5·1		0·719		2·9	2·8	4·6		<0·001		−1·6	−2·2	5·4		<0·001		−6·1	−7·0	9·0		<0·001	
B (*n* 150)	M	−7·4	−7·0	4·2	−0·1	<0·001	0·877	−1·5	−1·34	4·0	−1·5	<0·001	0·002	−8·9	−8·5	4·4	−1·3	<0·001	0·027	0·0	0·0	3·7	7·3	0·999	<0·001
I (*n* 150)		−7·3	−7·1	3·9		<0·001		−0·0	−0·2	4·3		0·922		−7·6	−7·5	5·7		<0·001		−7·3	8·1	8·5		<0·001	<0·001
B (*n* 150)	F	−5·4	−5·2	4·3	−1·4	<0·001	0·002	−3·2	−3·1	3·5	−1·4	<0·001	<0·001	−7·3	−6·7	5·4	−1·4	<0·001	<0·001	−7·1	−6·5	8·8	−7·1	<0·001	<0·001
I (*n* 150)		−4·0	−4·4	3·7		<0·001		−1·8	−1·9	3·4		<0·001	<0·001	−5·1	−5·4	4·1		<0·001	<0·001	0·0	0·1	3·3		0·996	<0·001
C (*n* 150)	M	−2·7	−2·8	5·0	4·6	<0·001	<0·001	5·0	4·6	5·6	5·0	<0·001	<0·001	−3·6	−4·2	6·8	5·0	<0·001	<0·001	20·5	21·3	8·7	27·8	<0·001	<0·001
I (*n* 150)		−7·3	−7·1	3·9		<0·001	<0·001	−0·0	−0·2	4·3		0·922	<0·001	−7·6	−7·5	5·7		<0·001	<0·001	−7·3	8·1	8·5		<0·001	<0·001
C (*n* 150)	F	−0·1	−0·8	5·1	3·9	0·719	<0·001	2·9	2·8	4·6	4·7	<0·001	<0·001	−1·6	−2·2	5·4	4·7	<0·001	<0·001	−6·1	−7·0	9·0	−6·1	<0·001	<0·001
I (*n* 150)		−4·0	−4·4	3·7		<0·001		−1·8	−1·9	3·4		<0·001		−5·1	−5·4	4·1		<0·001		0·0	0·1	3·3		0·996	

MD, mean difference; SS, stretch stature; TAS, total armspan; DSE, demi-span gender-specific equation; HAS, half-armspan; B, Black African; C, Caucasian; I, Indian; M, male; F, female.

* One-sample *t* test (two-tailed).

† Independent-samples *t* test (two-tailed).

When comparing Caucasians with Black Africans and Indians, there were significant differences (*P*<0·001) in SS minus TAS. However, Indian and Black African males had similar SS minus TAS (*P*=0·877), but significant differences were found between Black African and Indian females (*P*=0·002).

There were significant differences in SS minus WHO-adjusted equation (*P*<0·001) among the race and gender groups surveyed, except between Black African and Caucasian females (*P*=0·322). SS minus DS and SS minus HAS×2 differed significantly between all race and gender groups (*P*<0·001).

When the height estimation methods were compared with SS in Black African males, the methods that significantly (*P* <0·001) overestimated SS were HAS×2 (−8·9 (sd 4·4) cm), TAS (−7·4 (sd 4·2) cm) and DSE (−1·5 (sd 4·0) cm). The most predictive height estimation method for the calculation of true height was the WHO-adjusted equation (0·0 (sd 3·7) cm; *P*=0·999).

In Black African females, the height estimation methods that significantly (*P*<0·001) overestimated SS were the WHO-adjusted equation (−7·1 (sd 8·8) cm), TAS (−5·4 (sd 4·3) cm), HAS×2 (−7·3 (sd 5·4) cm) and DSE (−3·2 (sd 3·5) cm). The most predictive height estimation method for the calculation of true height was the DSE.

For Caucasian males, the height estimation methods that significantly (*P*<0·001) underestimated SS were the WHO-adjusted equation (20·5 (sd 8·7) cm), DSE (5·0 (sd 5·6) cm) and HAS×2 (5·0 (sd 5·6) cm). TAS (−2·7 (sd 5·0) cm) significantly (*P*<0·001) overestimated SS. The most predictive height estimation method for the calculation of true height was TAS.

In Caucasian females, the height estimation methods that significantly (*P*<0·001) underestimated SS were DSE (2·9 (sd 4·6) cm) and HAS×2 (2·9 (sd 4·6) cm). The WHO-adjusted equation (−6·1 (sd 9·0) cm) significantly (*P*<0·001) overestimated SS. The most predictive height estimation method for the calculation of true height was TAS (−0·1 (sd 5·1) cm; *P*=0·719).

For Indian males, the height estimation methods that significantly (*P*<0·001) overestimated SS were the WHO-adjusted equation (−7·3 (sd 8·5) cm), TAS (−7·3 (sd 3·9) cm) and HAS ×2 (−0·0 (sd 4·3) cm). The most predictive height estimation method for the calculation of true height was DSE (−0·0 cm (sd 4·3); *P*=0·922).

In Indian females, the height estimation methods that significantly (*P*<0·001) overestimated SS were HAS×2 (−5·1 (sd 4·1) cm), TAS (−4·0 (sd 3·7) cm) and DSE (−1·8 (sd 3·4) cm). The most predictive height estimation method for the calculation of true height was the WHO-adjusted equation (0·0 (sd 3·3) cm; *P*=0·996).

## Discussion

In South Africa, little is known about the relationship between height and arm-associated height estimation methods between gender and race groups^(^ ^11^ ^)^. Our results identified a strong relationship between SS measurements and the height estimates thereof, which was supported by the findings of the present study’s pilot study^(^ ^11^ ^)^. This suggests that the height estimation methods investigated in the current study could be used to calculate true height^(^ ^1^ ^,^ ^3^ ^)^.

### Race and gender

Strong relationships and correlations were identified between gender and height estimation measurements, with males having larger SS and arm-associated measurements than females, thereby supporting that males are anatomically larger than females^(^ ^11^ ^,^ ^18^ ^–^ ^21^ ^)^. Moreover, differences were identified between males and females within the same race group. Hence, these findings are indicative of the fact that gender-specific height estimation methods should be used when estimating height^(^ ^22^ ^–^ ^26^ ^)^.

Unlike gender, no significant associations were found between height estimation measurements and race. However, significant differences were documented when study participants were stratified according to race and gender. The latter was similar to findings reported by several other studies^(^ ^22^ ^–^ ^26^ ^)^. Caucasians differed from Black Africans and Indians in terms of height estimation measurements, whereas there were similarities between these height estimation measurements for Black Africans and Indians. Therefore, it is evident that Caucasians are anatomically different in comparison to the other race groups by both SS and arm-associated measurements. However, Indians and Black Africans are similar in terms of all arm-associated measurements, especially when comparing males of both races. When females were compared, anatomical similarities were identified for the SS measurement and TAS measurement between Black African and Indian females, as well as anatomical similarities for arm-associated measurements between Black African and Caucasian females.

### Half-armspan ×2

Significant differences were identified in the comparison of HAS×2 and SS for all race groups of both genders^(^ ^11^ ^)^. Furthermore, HAS×2 overestimated SS in the calculation of true height for all race groups of both genders^(^ ^11^ ^)^. Therefore, it is possible that anatomical variations exist in the HAS measurement for the study participants in terms of race as well as between genders.

### Demi-span equation

Significant differences were identified in the comparison of DSE and SS between all race groups of both genders^(^ ^11^ ^)^. Therefore, it is possible that anatomical variations exist in the DS measurement for the study participants’ race groups and between genders.

### The WHO equation

Significant differences were found in the comparison of the WHO equation and SS between all race groups of both genders^(^ ^11^ ^)^. The WHO^(^ ^17^ ^)^ recommends that the WHO equation should be used as a universal equation to calculate estimated height. However, in the current study the WHO equation was identified to be the least predictive height estimation method in the calculation of true height as it statistically significantly underestimated height for all race groups of both genders^(^ ^11^ ^,^ ^27^ ^)^. On the other hand, the WHO-adjusted equation agreed sufficiently with SS and was predictive of true height in the study sample, especially Black African males and Indian females.

### Total armspan

For the study sample, anatomical variations existed in the TAS measurement in relation to race and between genders. However, Black Africans and Indians had similar TAS measurements. Overall, TAS overestimated height^(^ ^3^ ^,^ ^4^ ^,^ ^11^ ^,^ ^15^ ^,^ ^18^ ^,^ ^19^ ^,^ ^22^ ^–^ ^24^ ^,^ ^26^ ^,^ ^28^ ^–^ ^35^ ^)^ in all race groups of both genders.

### The maximal height theory

Anatomical variation was a common trend within the study sample, where in this instance adult height was reflective of a collection of determining height factors unique to each population^(^ ^36^ ^)^. For the purpose of the current paper, maximal height refers to the optimum height that one is genetically programmed for, irrespective of the effects of age or morbidity. This can collectively be referred to as an individual’s maximal genetic growth potential (see online supplementary material, Supplemental Fig. 10). Therefore, the ability to achieve maximal height is dependent on the exposure to growth-determining factors such as genetics, nutrition, maternal health and nutrition during pregnancy, socio-economic status and the environment. Furthermore, within populations, these factors may lead to secular change^(^ ^37^ ^,^ ^38^ ^)^, which is the accumulative and repetitive biological changes that may occur within an individual to give rise to an effect; in this case, an increase or decrease in stature as well as variability in arm-associated measurements.

### The Vitruvius theory

The maximal height theory highlights the possible factors that determine adult height, whereas the Vitruvius theory identifies the possible correlation between maximal height and TAS. When Marcus Vitruvius Pollio first described the body as a circle within a square^(^ ^39^ ^)^, he was ascribing to the ideal that body dimensions are made up of exactly similar parts around an axis such as the navel (see online supplementary material, Supplemental Fig. 11). His depiction of body symmetry led to the understanding that human height would be equal to TAS. The current study findings are in accordance with those of several studies that have found TAS does not equate to SS and overestimates SS^(^ ^3^ ^,^ ^11^ ^,^ ^15^ ^,^ ^18^ ^,^ ^19^ ^,^ ^22^ ^–^ ^24^ ^,^ ^26^ ^,^ ^28^ ^–^ ^35^ ^)^.

However, it has been documented that this Vitruvius phenomenon can occur within a population^(^ ^40^ ^,^ ^41^ ^)^. According to the current study’s findings, the most likely race group for this phenomenon to occur in would be Caucasians. Although statistically significant differences were identified between SS and TAS among Caucasian males (−2·7 (sd 5·0) cm; *P*<0·001), no significant differences were identified between SS and TAS in Caucasian females (−0·1 (sd 5·1) cm; *P*=0·719). As a result, the Vitruvius phenomenon is more likely to occur within Caucasian females than Caucasian males.

Furthermore, if TAS is found to equate to SS, this implies that the study participant was exposed to positive secular growth conditions which were consistently available between conception and the first 2 years of life (the window period) and beyond. These secular growth conditions have a potential impact on the leg long bones, especially the tibula and fibula (see online supplementary material, Supplemental Fig. 12). Negative secular growth conditions may cause the difference between maximal height and actual skeletal height to increase and as a result cause a potential decrease in long bone length, i.e. decrease of height measurement. On the other hand, positive secular growth conditions may cause the difference between maximal height and actual skeletal height to decrease and as a result cause a potential increase in long bone length, i.e. increase of height measurement. Therefore based on the present study’s findings, it is hypothesized that TAS represents the participant’s maximal height or genetically intended height.

### Public health relevance

Anthropometric measurements, in this case height, form part of health surveillance, clinical investigations and growth monitoring, which eventually translate into public health intervention and/or individual treatment. The present study’s findings (summarized in Table 4) would be applicable for use in young South African adults aged <30 years. Height loss begins from the age of 30 years^(^ ^42^ ^)^ and consequently these findings may not be relevant to those aged >30 years.

**Table 4:** Arm-associated height estimation methods that are most predictive of stretch stature, according to race and gender, among a convenience sample of young South African adults aged 18–24 years, KwaZulu-Natal, 2015

Race	Gender	Arm-associated height estimation equation*
B	M	Height (cm) = {[0·73×(2×right half-armspan (cm))+0·43]+39·1246}
	F	Height (cm) = [(1·35×right demi-span (cm))+60·1]
C	M	Height (cm) = (total armspan (cm))
	F	Height (cm) = (total armspan (cm))
I	M	Height (cm) = [(1·40×right demi-span (cm))+57·8]
	F	Height (cm) = {[0·73×(2×right half-armspan (cm))+0·43]+38·7598}

B, Black African; C, Caucasian; I, Indian; M, male; F, female.

* Height estimates measured in centimetres.

### Limitations and strengths

Time and financial constraints prevented the inclusion of a larger sample size, which would have allowed for the validation of the arm-associated height estimation methods for use in young South African adults. The current study did not include different age categories, which would have accounted for potential age-related height loss^(^ ^42^ ^)^. There was a lack of local South African-based anthropometric studies which have investigated the use of arm-associated height estimation methods for the calculation of true height in young South African adults.

A strength of the present study is that its findings have contributed to the development of new anthropometric methodology. It is the first African anthropometric study, to our knowledge, that has identified the importance of population-specific anthropometric methods. The only other African studies to undertake similar analyses were a study investigating knee height in the elderly^(^ ^21^ ^)^, an Ethiopian study^(^ ^24^ ^)^, a Nigerian study^(^ ^31^ ^)^ and the present study’s pilot study^(^ ^11^ ^)^.

## Conclusion

Height is a basic anthropometric measurement which can be calculated through using arm-associated measurements. Although universal equations for estimating height exist such as the WHO method^(^ ^17^ ^)^, there is an increased need to use methods that are population-specific, as well as gender- and race-specific.

## Supplementary material

For supplementary material accompanying this paper visit https://doi.org/10.1017/S1368980016002330. click here to view supplementary material
