# Supplementary material for: Estimation of true height: a study in population-specific methods among young South African adults
Source: Public Health Nutr. 2016 Sep 9;20(2):210–9. doi: 10.1017/S1368980016002330 (PMC5244443; doi:10.1017/S1368980016002330)
Supplement: Supplementary file 1 [file S1368980016002330sup.zip › S1368980016002330sup012.pdf]

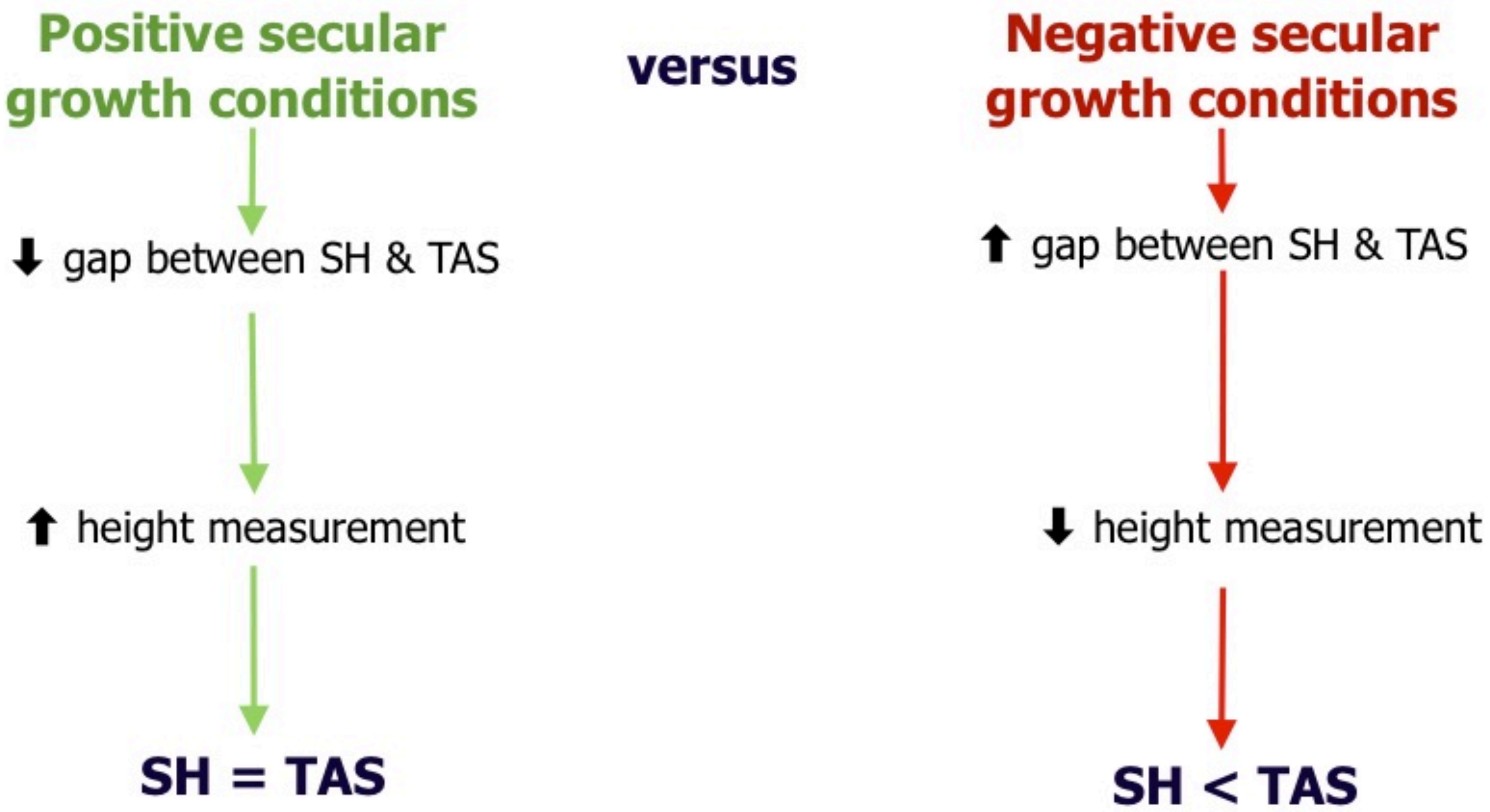

Supplemental Figure 12: The Vitruvius theory
