# Supplementary figures and images for: Estimation of true height: a study in population-specific methods among young South African adults
Source: Public Health Nutr. 2016 Sep 9;20(2):210–9. doi: 10.1017/S1368980016002330 (PMC5244443; doi:10.1017/S1368980016002330)

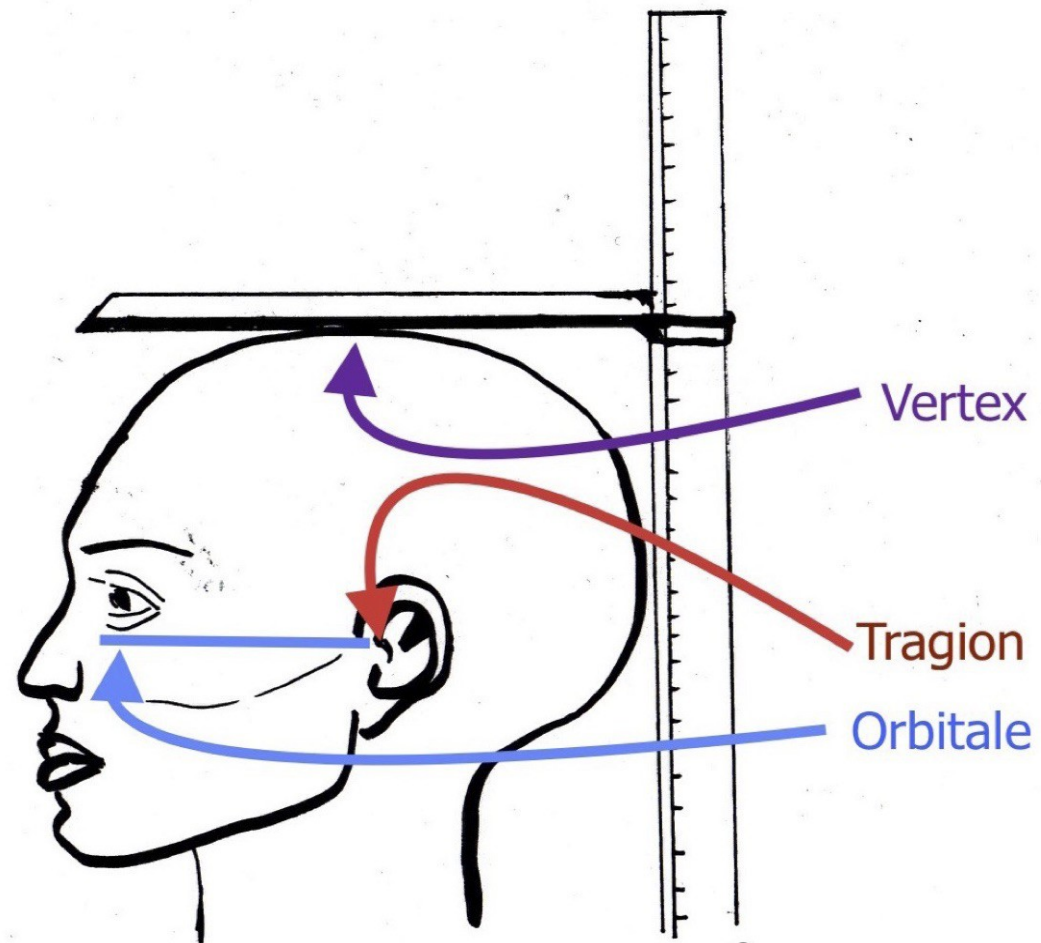

**Supplemental Figure 2:** The head positioned in the Frankfort plane

Supplement: Supplementary file 1 [file S1368980016002330sup.zip › S1368980016002330sup002.pdf]

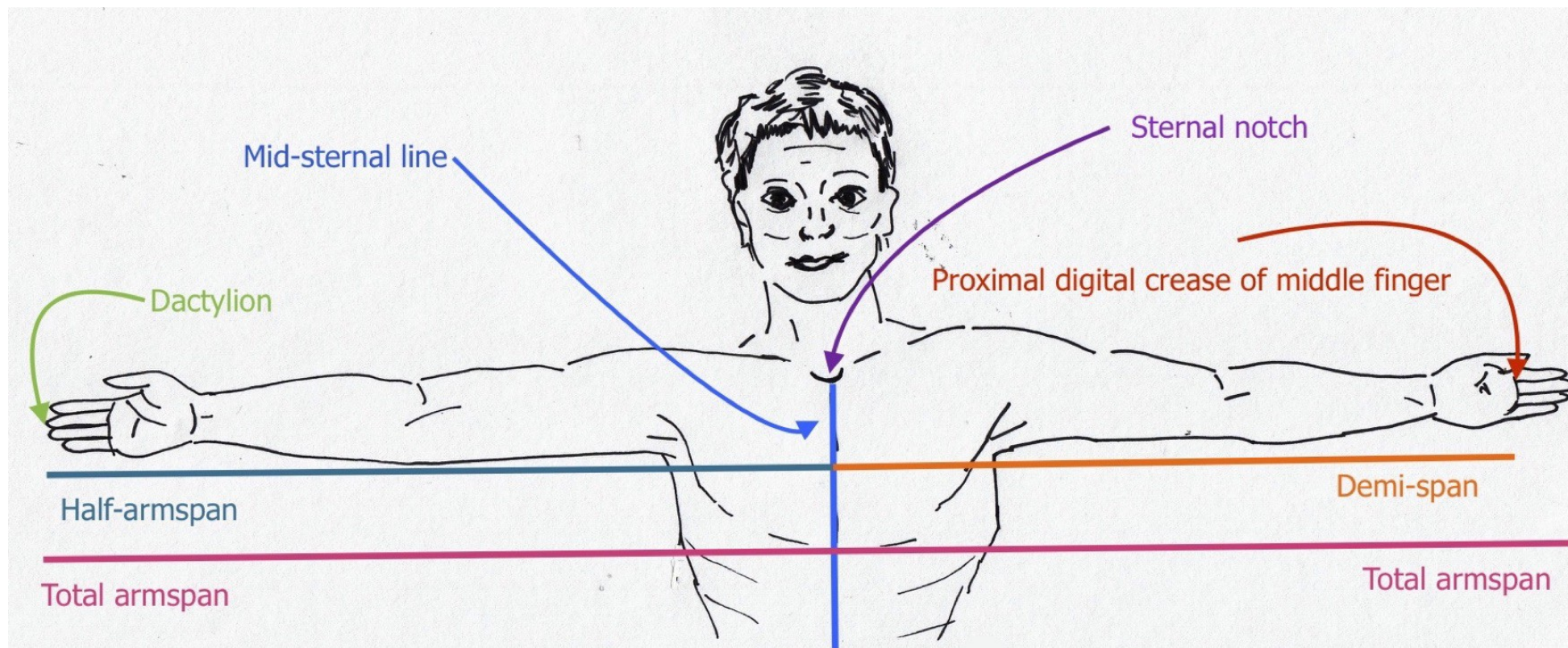

**Supplemental Figure 3:** Arm-associated height estimation methods, with measurement landmarks

Supplement: Supplementary file 1 [file S1368980016002330sup.zip › S1368980016002330sup003.pdf]

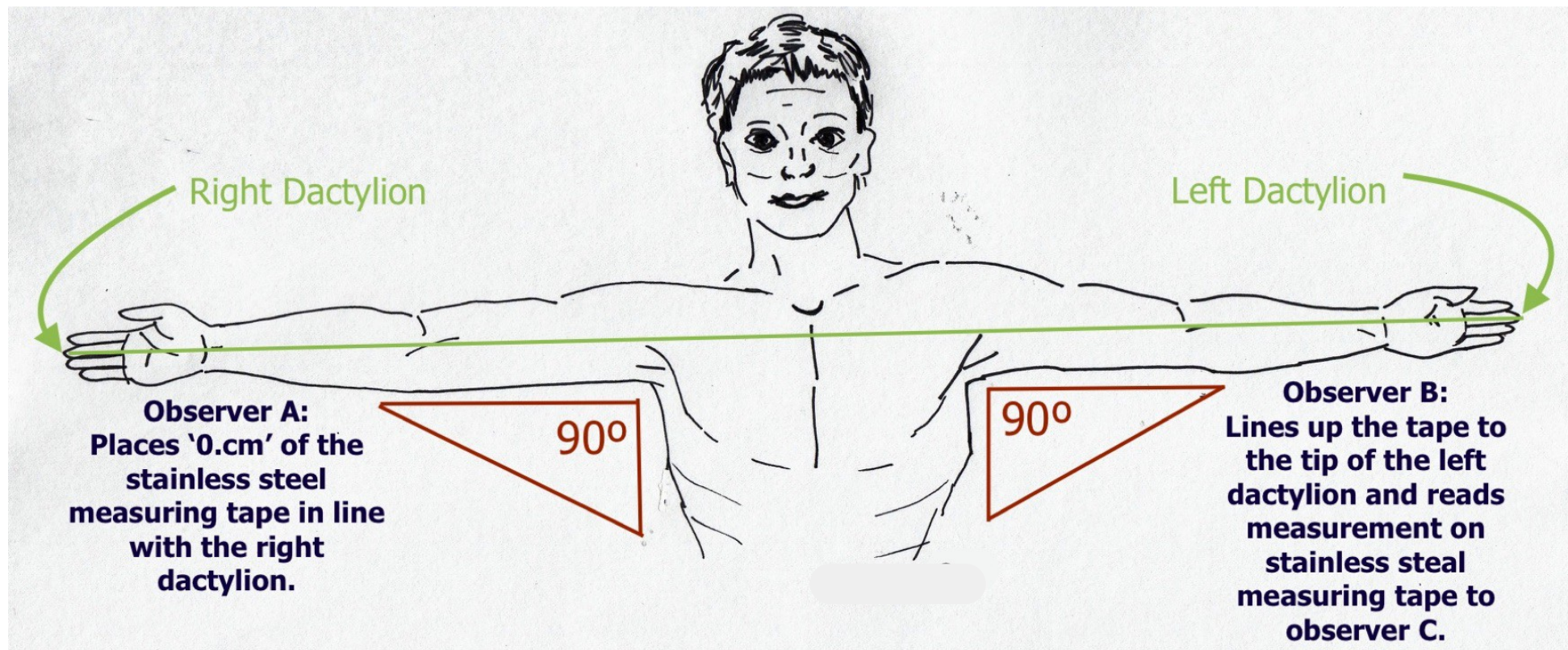

**Supplemental Figure 4:** Measurement of total armspan

Supplement: Supplementary file 1 [file S1368980016002330sup.zip › S1368980016002330sup004.pdf]

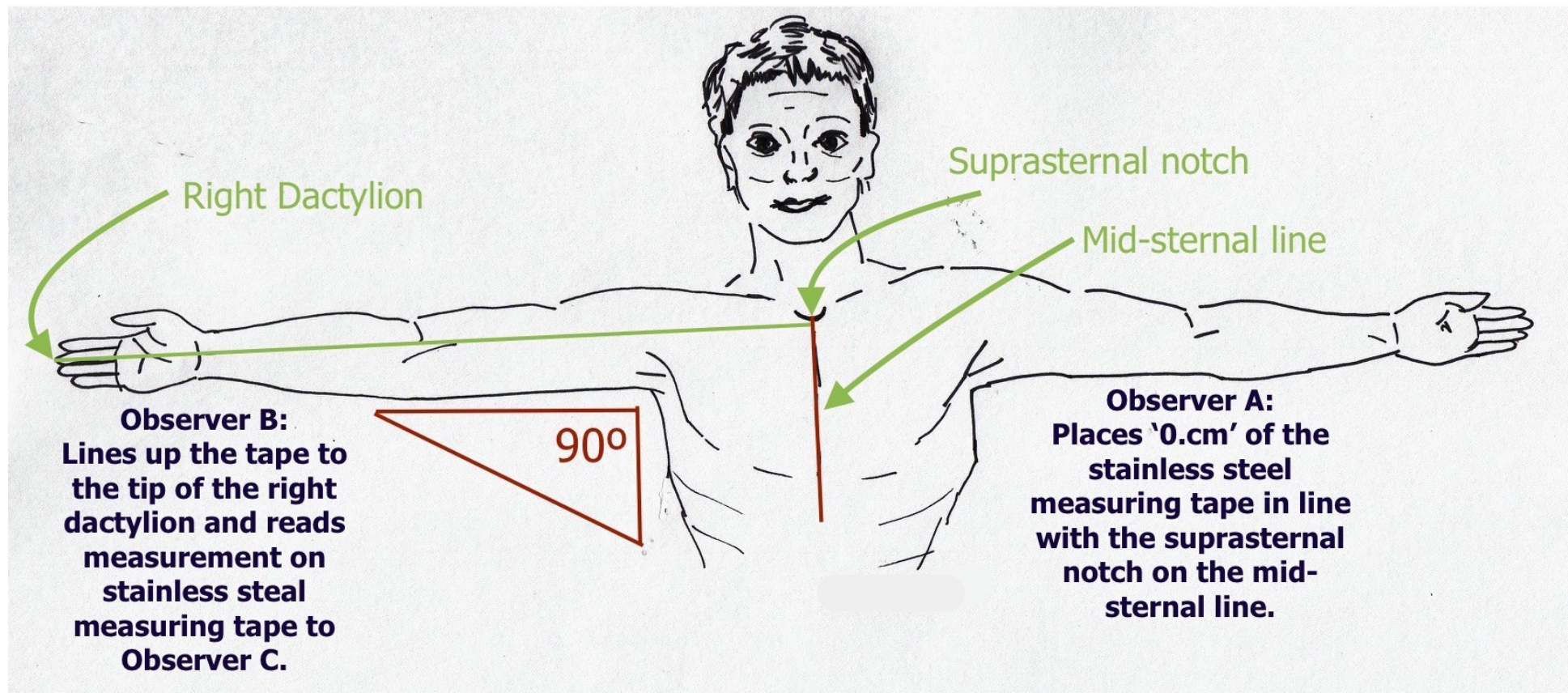

**Supplemental Figure 5:** Measurement of half-armspan

Supplement: Supplementary file 1 [file S1368980016002330sup.zip › S1368980016002330sup005.pdf]

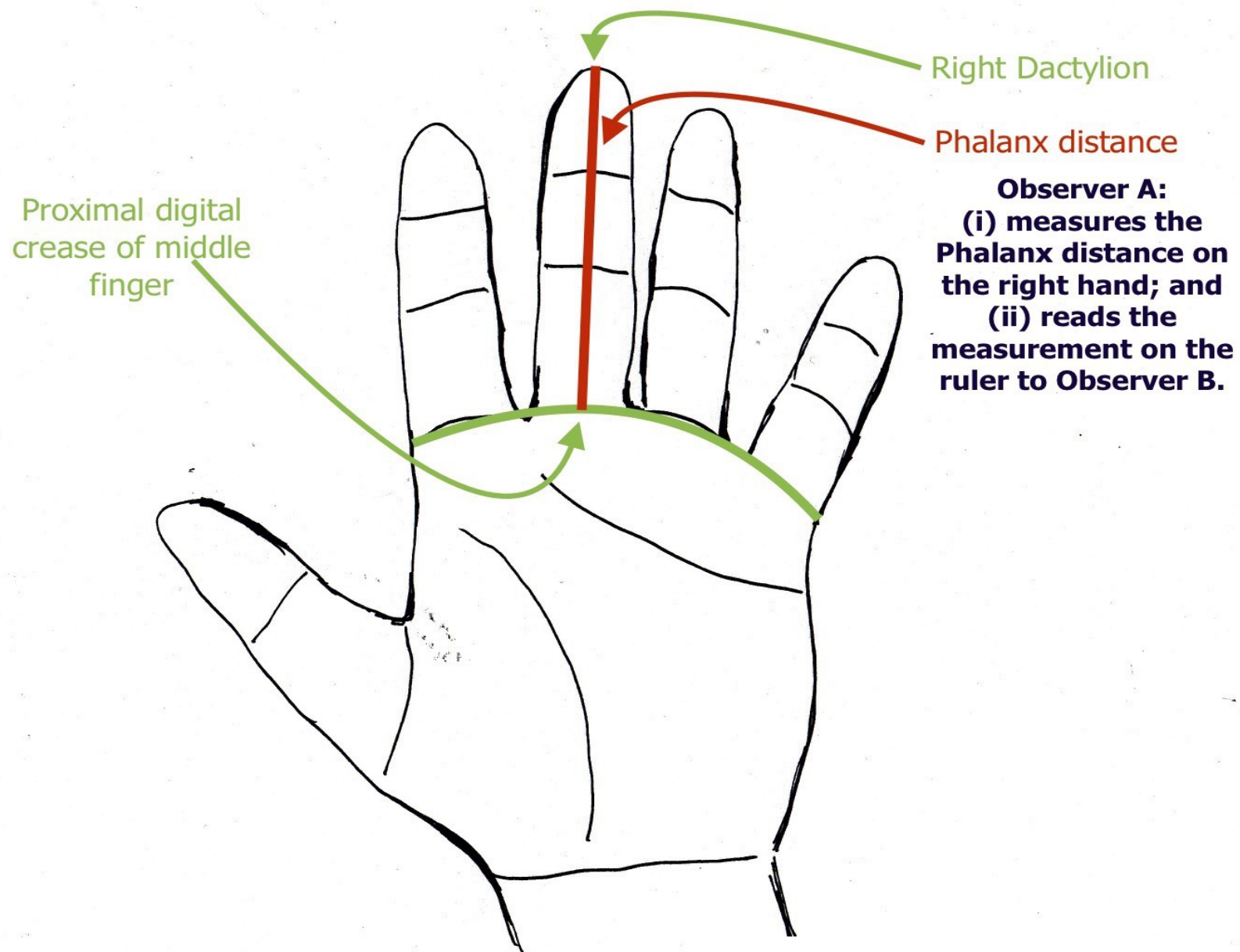

**Supplemental Figure 6:** Alternative methods to measure demi-span, with anatomical landmarks

Supplement: Supplementary file 1 [file S1368980016002330sup.zip › S1368980016002330sup006.pdf]

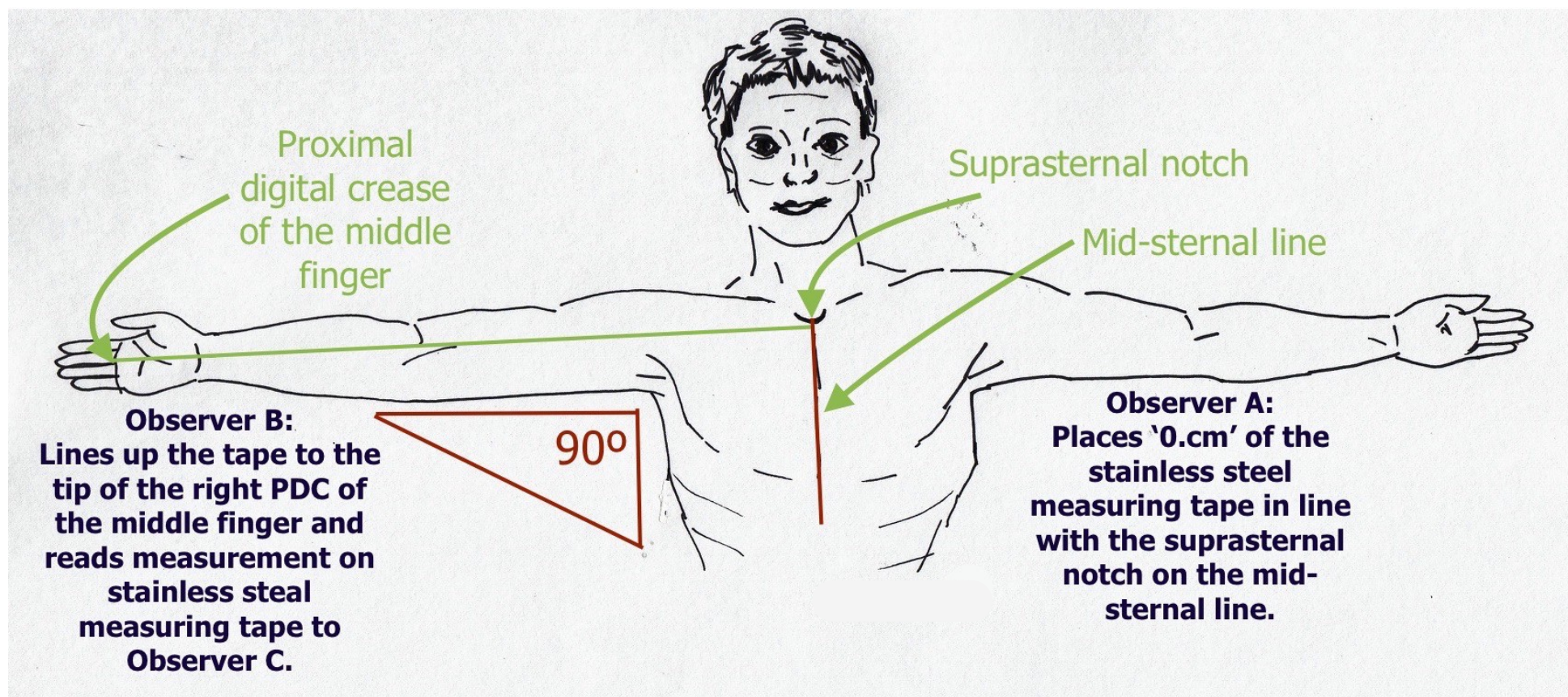

**Supplemental Figure 7:** Measurement of demi-span measurement, using the original method

Supplement: Supplementary file 1 [file S1368980016002330sup.zip › S1368980016002330sup007.pdf]

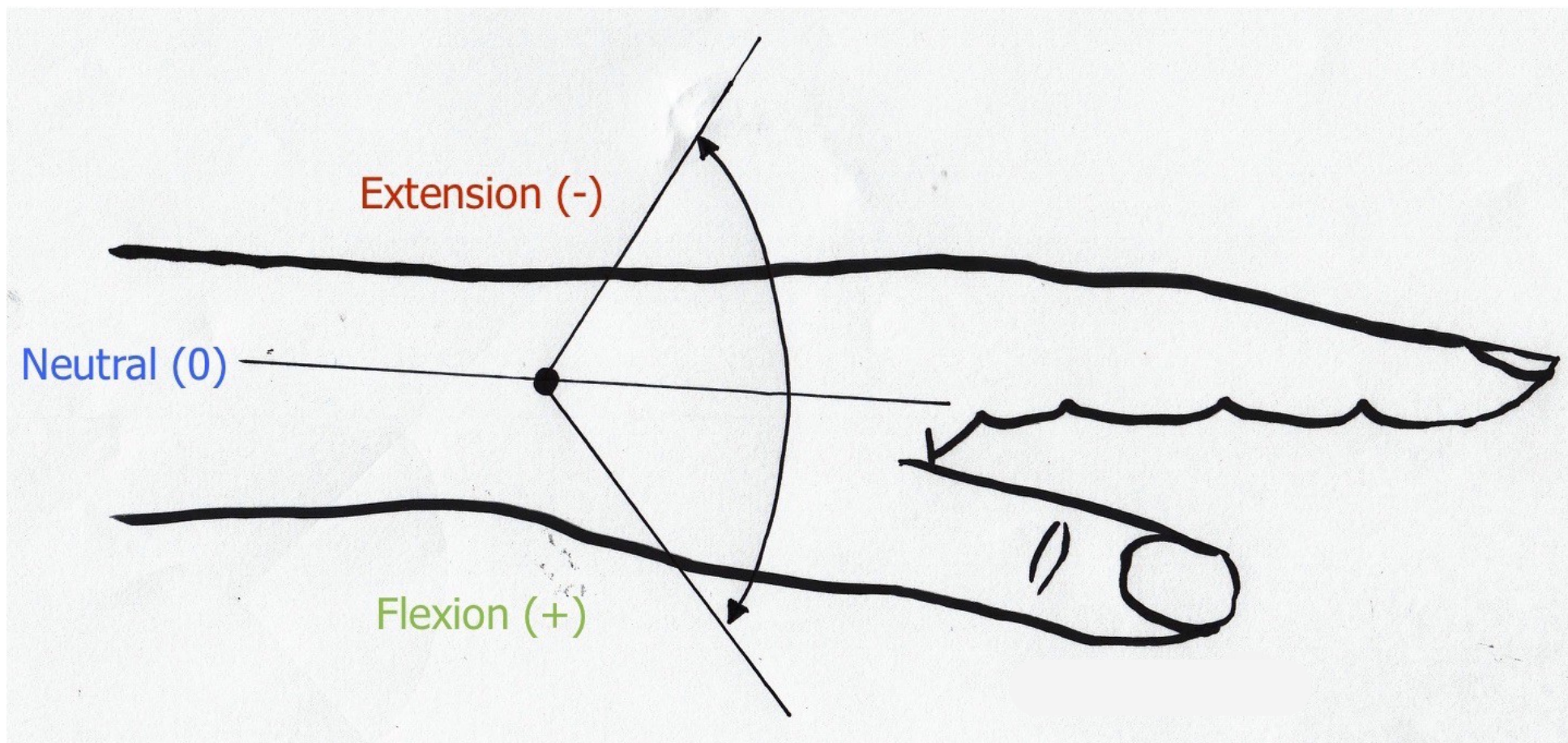

**Supplemental Figure 8:** The wrist in neutral A position

Supplement: Supplementary file 1 [file S1368980016002330sup.zip › S1368980016002330sup008.pdf]

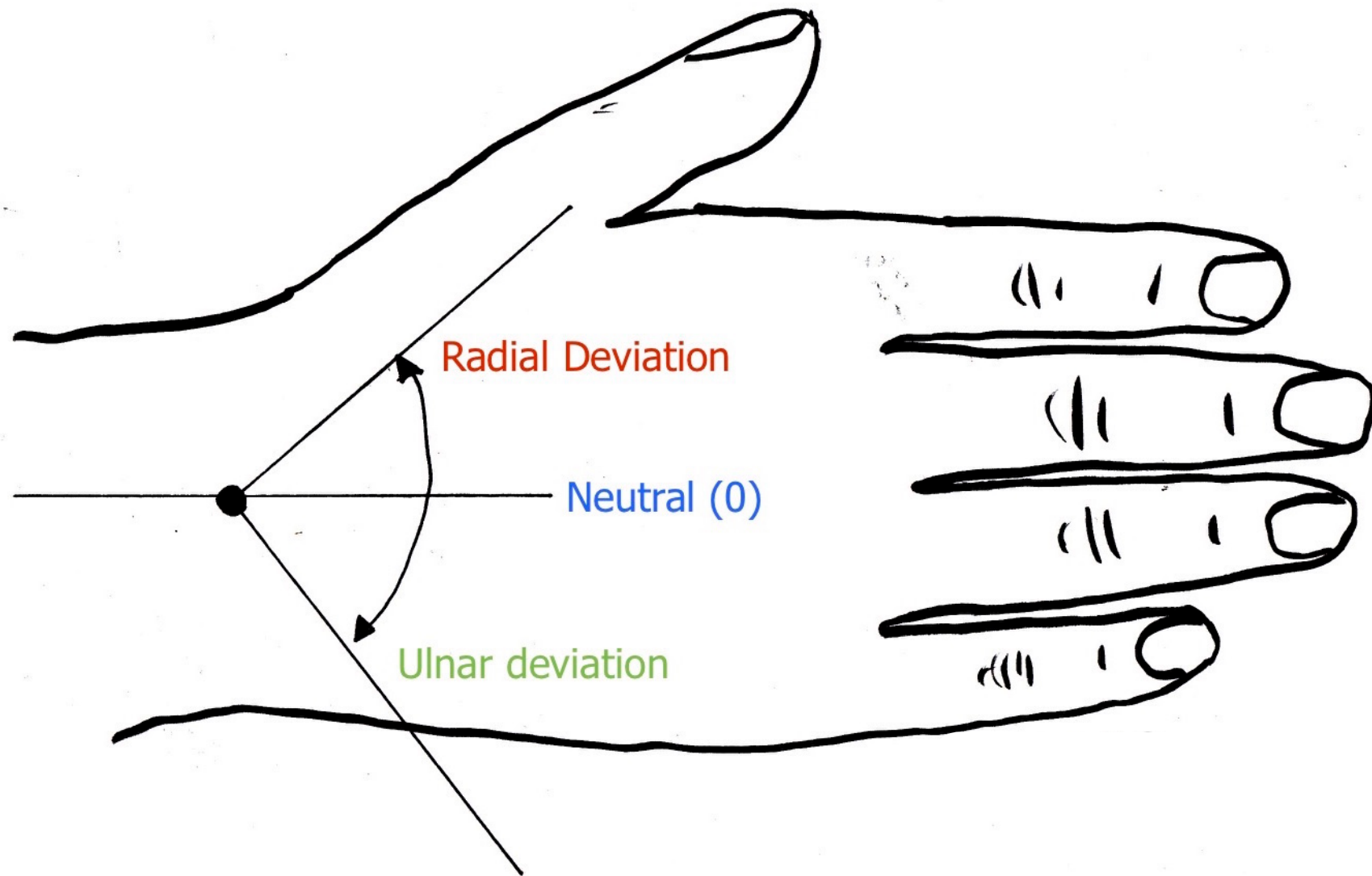

**Supplemental Figure 9:** The wrist in a neutral B position

Supplement: Supplementary file 1 [file S1368980016002330sup.zip › S1368980016002330sup009.pdf]

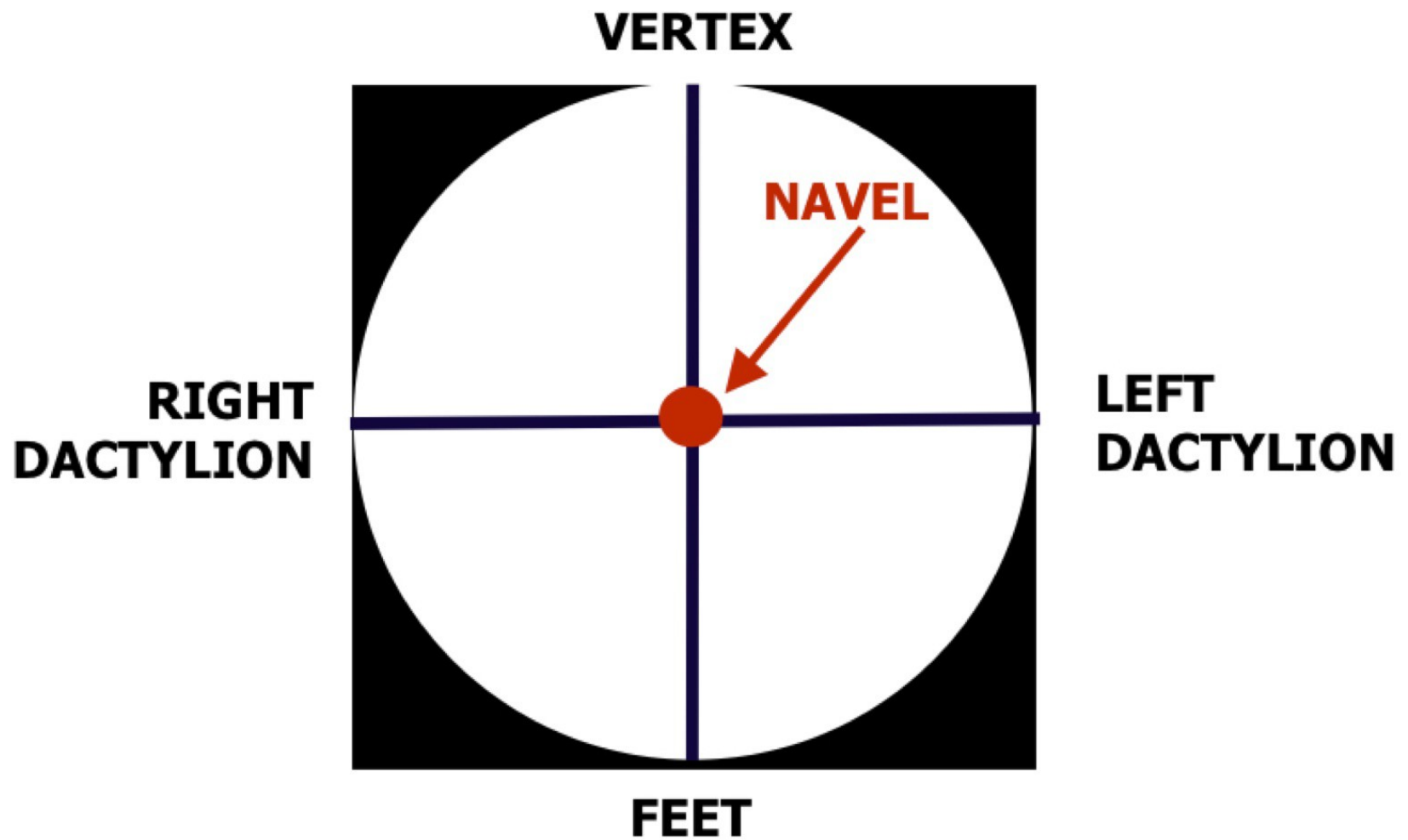

**Supplemental Figure 11:** Diagrammatic illustration of man, based on the Vitruvius' ideology

Supplement: Supplementary file 1 [file S1368980016002330sup.zip › S1368980016002330sup011.pdf]
