# Supplementary material for: Estimation of true height: a study in population-specific methods among young South African adults
Source: Public Health Nutr. 2016 Sep 9;20(2):210–9. doi: 10.1017/S1368980016002330 (PMC5244443; doi:10.1017/S1368980016002330)
Supplement: Supplementary file 1 [file S1368980016002330sup.zip › S1368980016002330sup013.doc]

**Supplemental Tables**

**Supplemental Table 1:** Correlation between stretch stature and height estimation methods thereof

| Race, gender & SS |  | SS & height estimations thereof |  | r value |  | p value** |
| --- | --- | --- | --- | --- | --- | --- |
| Stretch Stature |  | Total arm-span |  | 0.887 |  | < 0.001 |
|  |  | Demi-span |  | 0.843 |  | < 0.001 |
|  |  | Demi-span equation |  | 0.851 |  | < 0.001 |
|  |  | Half-arm span |  | 0.856 |  | < 0.001 |
|  |  | Half-arm span × 2 |  | 0.856 |  | < 0.001 |
|  |  | WHO-equation |  | 0.856 |  | < 0.001 |
|  |  | WHO-adjusted equation |  | 0.186 |  | < 0.001 |
| ** Pearson’s correlation coefficient. Correlation is significant at the 0.01 level (2-tailed)  SS= Stretch Stature | | | | | | |

**Supplemental Table 2: WHO-adjusted equations according to race and gender**

| Race |  | Gender |  | Height estimation equations^#^ |
| --- | --- | --- | --- | --- |
| Black African |  | Male |  | Height (cm) = [0.73 × (2 × right half-armspan(cm)) + 0.43) + 39.1246] |
|  |  | Female |  | Height (cm) = [0.73 × (2 × right half-armspan(cm)) + 0.43) + 37.1299] |
| Caucasian |  | Male |  | Height (cm) = [0.73 × (2 × right half-armspan(cm)) + 0.43) + 45.3405] |
|  |  | Female |  | Height (cm) = [0.73 × (2 × right half-armspan(cm)) + 0.43) + 43.1595] |
| Indian |  | Male |  | Height (cm) = [0.73 × (2 × right half-armspan(cm)) + 0.43) + 40.4746] |
|  |  | Female |  | Height (cm) = [0.73 × (2 × right half-armspan(cm)) + 0.43) + 38.7598] |
| # Standing height calculated in centimetres (cm) | | | | |
